# Supplementary material for: Prediction of Extraintestinal Manifestations in Inflammatory Bowel Disease Using Clinical and Genetic Variables with Machine Learning in a Latin IBD Group
Source: Int J Mol Sci. 2025 Jun 15;26(12):5741. doi: 10.3390/ijms26125741 (PMC12192962; doi:10.3390/ijms26125741)
Supplement: Supplementary file 1 [file ijms-26-05741-s001.zip › ijms-3627693-supplementary.pdf]

Supplementary Tables

Supplementary Tables S1, S2, and S3. Genetic variants significantly associated with EIMs.

Tables S1, S2, and S3 present data on single nucleotide polymorphisms (SNPs) identified as significantly associated with extraintestinal manifestations (EIMs) after adjustments for ancestry, sex, and age. The data is displayed in a structured format, including detailed statistical metrics such as odds ratios (OR), confidence intervals (CI), and P-values. Significant P-values are highlighted in bold to enhance readability.

The analysis involved three genotypes, recessive, dominant, and additive models. However, only models that yielded statistically significant results for each SNP are included in these tables. The reference genotype serves as the basis for comparison. Ref:Reference genotype for comparison.

Table S1

**rs4410871**	**EIMs**		**Univariate**			**Adjusted by Ancestry, Sex, and Age**		
**Three genotypes**	**EIMs-=149**	**EIMs+=83**	**OR**	**CI**	**P-Value**	**OR**	**CI**	**P-Value**
CC	72	30	Ref	Ref	Ref	Ref	Ref	Ref
TC	57	46	1.94	1.09-3.47	0.03	1.99	1.11-3.65	**0.02**
TT	20	7	0.84	0.30-2.12	0.72	0.94	0.33-2.43	0.90
**Additive**	**EIMs-=149**	**EIMs+=83**	**OR**	**CI**	**P-Value**	**OR**	**CI**	**P-Value**
CC =0	72	30	Ref	Ref	Ref	Ref	Ref	
TC =1	57	46	1.94	1.09-3.47	0.03	1.99	1.11-3.65	**0.02**
TT =2	20	7	0.84	0.30-2.12	0.72	0.94	0.33-2.43	0.90

Table S2

rs3132680	EIMs		Univariate			Adjusted by Ancestry, Sex, and Age		
**Recessive**	**EIMs-=149**	**EIMs+=83**	**OR**	**CI**	**P-Value**	**OR**	**CI**	**P-Value**
CA/CC	135	68	Ref	Ref	Ref	Ref	Ref	Ref
AA	14	15	2.13	0.97-4.71	0.06	2.55	1.12-5.89	**0.03**
**Three genotypes**	**EIMs-=149**	**EIMs+=83**	**OR**	**CI**	**P-Value**	**OR**	**CI**	**P-Value**
AA	14	15	Ref	Ref	Ref	Ref	Ref	Ref
CA	60	19	0.30	0.12-0.72	0.01	0.23	0.09-0.58	**0.002**
CC	75	49	0.61	0.27-1.38	0.23	0.51	0.21-1.18	0.12
**Additive**	**EIMs-=149**	**EIMs+=83**	**OR**	**CI**	**P-Value**	**OR**	**CI**	**P-Value**
CC=0	75	49	Ref	Ref	Ref	Ref	Ref	Ref
CA=1	60	19	0.48	0.25-0.90	0.02	0.45	0.23-0.85	**0.02**
AA=2	14	15	1.64	0.73-3.73	0.23	1.98	0.85-4.68	0.12

rs3823417	EIMs		Univariate			Adjusted by Ancestry, Sex, and Age		
**Dominant**	**EIMs-=149**	**EIMs+=83**	**OR**	**CI**	**P-Value**	**OR**	**CI**	**P-Value**
GG	76	52	Ref	Ref	Ref	Ref	Ref	Ref
AG/AA	73	74	2.14	0.79-5.93	0.13	0.56	0.32-0.99	**0.05**
**Three genotypes**	**EIMs-=149**	**EIMs+=83**	**OR**	**CI**	**P-Value**	**OR**	**CI**	**P-Value**
AA	8	9	Ref	Ref	Ref	Ref	Ref	Ref
AG	65	22	0.30	0.10-0.88	0.03	0.30	0.10-0.90	**0.03**
GG	76	52	0.61	0.22-1.69	0.34	0.66	0.22-1.99	0.44
**Additive**	**EIMs-=149**	**EIMs+=83**	**OR**	**CI**	**P-Value**	**OR**	**CI**	**P-Value**
GG	76	52	Ref	Ref	Ref	Ref	Ref	Ref
AG	65	22	0.49	0.27-0.89	0.02	0.45	0.24-0.83	**0.01**
AA	8	9	1.64	0.59-4.65	0.34	1.52	0.53-4.48	0.44

Table S3
